# The Mechanism of *CP fandom* Behaviors among Chinese Young Adults: A Grounded Theory Study

**DOI:** 10.3390/bs13010030

**Published:** 2022-12-29

**Authors:** Wanqi Zhou, Yueling Zhang, Yuner Li, Qingyang Sun, Zeyang Yang

**Affiliations:** 1 Department of Psychology, School of Education, Soochow University, Suzhou 215123, China; 2 Department of Applied Linguistics, Xi’an Jiaotong-Liverpool University, Suzhou 215123, China

**Keywords:** shipping behaviors, fandom, CP fans, grounded theory study

## Abstract

*CP fandom* behaviors or *shipping*, a growing popular phenomenon among Chinese young adults, refers to the activities of fans who take great satisfaction from the romantic relationships and interactions of their preferred pairings of idols or virtual characters. *CP fans* are regarded as a special group of fans with unique identities and interaction styles. This grounded theory study was conducted to explore the mechanism of *CP fandom* behaviors. Semi-structured in-depth interviews were conducted with thirty-one Chinese *CP fans* (twenty-eight females and three males). The antecedents, development, behavioral patterns, and consequences of *shipping* were identified in a comprehensive model. The reasons for *CP fandom* behaviors include individual factors (e.g., psychological projection, compensation, and social needs) and external factors (e.g., pop culture and internet environment). The consequences include positive emotional experiences, changed love values, and improved social interaction. *CP fandom* behaviors can be different in terms of the fans’ degrees of engagement, and the development of *shipping* can be divided into three stages: exploratory stage, formation and stability stage, and rupture stage. This study contributes to the literature of *CP fandom* behaviors among young adults in China and proposes the directions of future studies on such topics.

## 1. Introduction

*CP fandom behaviors* (*Ke CP* or *shipping*) are a special behavior of idolatry that has gradually become popular among young people in China in recent years. *CP* is the abbreviation of the English word *coupling* (pairing). It was originally used in the context of virtual fictions (in the forms of comics, animation, and video games), but later, it extended to all character pairing relationships, including in the real world [1]. *Ke CP* means fans take great satisfaction from the intimate relationships and interactions of their preferred pairings as if they were addicted to drugs (the verb *ke* in informal Chinese means being addicted to drugs) [2]. Such fans are thus termed *CP fans*.

*CP fans* not only have the characteristics of general fan groups but also have unique characteristics. The nature of celebrity *CP fandom* is not simply the adoration of the celebrity but an adoration of the real or imagined love relationship between idols [3]. *CP fans* are keen to match virtual characters or real celebrities and fantasize that they have some intimate relationship; they may have emotional identification and involvement with one or more pairs of couples within a certain period of time [4]. In this study, *CP fans* are defined as individuals who are obsessed with the intimate relationship between characters, which can be based on reality/facts or fan groups’ imagination; the characters can be virtual or real. In the English context, *shipping* refers to the act of pairing two characters into a romantic relationship, which corresponds to the Chinese term *Ke CP,* and the *shipper* is the subject of this act [5].

Many recent indents involving *CP fandom* in China have attracted wide public attention on social media, which are often associated with negative consequences. For example, the *227 incident* surrounding a pop idol *CP* (“Wang and Xiao”) re-creation work (fan fiction) has caused viral conflicts among fan groups, which had a huge negative social influence [6]. The number of this *CP*’s fans on Weibo (a Chinese well-known social media service) has reached more than 3 million (2022 data), which shows that *shipping* has long-lasting attraction and influence on fans. Growing up as digital natives, Chinese young adults are largely involved in *CP fandom* behaviors, which needs more investigation, especially on the psychological mechanism.

However, at present, research in this area in China is still scarce, and there is little evidence about the psychological mechanism of *CP fandom* behaviors. Therefore, this study takes Chinese *CP fans* as research objects and analyzes their characteristics and the reasons, development, and consequences of their *shipping/CP fandom* behaviors through qualitative approaches.

## 2. Literature Review

### 2.1. Identity and Individualization

Identity can be divided into individual identity and group identity. The former is constructed based on the unique quality of the individual, and the latter is formed based on the qualifications of group members [7]. Social identity refers to an individual’s recognition that he or she belongs to a particular social group, as well as the emotional and value significance of being a member of the group [8]. Identity theory holds that individuals tend to act in the way they like and are accepted by others, so they tend to play a certain role and internalize their identification with the role they play in their identity [9]. This theory helps to understand how *CP fans* behave and why they behave in certain ways. Therefore, the process of idolatry can be understood as a series of behaviors by fans in order to maintain and develop their fan identity [10].

The theory of individualization put forward by Ulrich Beck suggests that individualization removes people from traditional roles and constraints in many ways [11]. Yan pointed out that Chinese modern society allows a large number of personal choices, the non-standardization of personal development, and lifestyle politics [12]. China’s unique and novel consumer culture and sexual revolution have led to the rise of the pursuit of personal desires and emotional satisfaction. Under the background of individualization, Chinese young people’s behavior is characteristic of its time. Studying *shipping* from the perspective of individualization can help to better understand the psychological characteristics of contemporary youth and explore the impact of the social environment on them.

Fandom identity, as one specific social identity among young adults and teenagers, has been widely investigated in different contexts and proved to be complex [13,14,15,16]. Empirical studies suggest that fandom identity can be beneficial to individuals in fan groups. For example, fans can gain expected social status and a sense of belonging while participating in fandom activities [14]. Young fans could be encouraged by their idols and overcome difficulties in daily life [15]. However, being involved in fandom activities also has negative effects. For example, social conflicts can exist among certain fan groups of pop music celebrities when they compete with others, and fans try to validate their authentic fan identities in the community [16]. It thus seems necessary to further explore the impacts of fandom behaviors, especially among the emerging group of *CP fans*.

### 2.2. Fan Interaction

Empirical studies found that fan interaction is a recurrent theme of *shipping* behaviors. Williams explored the fans of the TV drama, *The West Wing*, and focused on how fans engage with the relationships between the characters [17]. It was found that the fans debate around gender and sexual issues in real life through *shipping* the characters and expressing their own identities. Thus, *CP fans* could share their thoughts on topics such as feminism or gender politics through *shipping* behaviors. Gao believes that the behavior of *shipping* is motivated by the need for debating about intimate relationships, self-satisfaction, and reassurance needs, especially among female *CP fans* [18].

Different types of fan groups may have different attitudes towards *CP*. Song explains that *shippers* (*CP fans*) who adore the relationship between two idols and *solo fans* who only adore one single idol have frequent conflicts with each other due to their different attributes as fans [19]. Such conflicts between fan groups can cause larger social conflicts on the internet, for example, the *227 incident*, as mentioned previously. Bothe analyzed the posts and comments of two *shipper* groups involving the same character and found that different fan groups had very different understandings of the relationships between the characters, and the two groups had constant conflicts [5].

### 2.3. Cultural Consumption and Dissemination

*Shipping* behaviors were reported to be linked with the consumption and dissemination of culture. Jenkins believes that fan culture is a multidimensional phenomenon with various components, which allows different forms of participation. As an important way of reflecting fan identity, fans could produce different types of *products* such as fandom-related writings (e.g., fictions or blogs) or videos [20]. Fans could obtain emotional satisfaction and a sense of belonging through reading or consuming the outputs of other community members [20]. Coleman regards *slash fiction* (fan fiction on imagined pairings of the same sex) as a kind of cultural appropriation, which is not immoral but creates a virtual space for imagination [21]. The culture here probably refers to the subcultures among young adults or specific groups such as *CP fans*. Smutradontri and Gadavanij believe that fans create texts as a way to show dedication and enthusiasm to their fan group [22]. These created texts or outputs can be transmitted through social media platforms which can reinforce the dissemination of their *CP fandom* culture. Ren points out that *shipping* behaviors on social media have been one important part of media participation among the young generation [4]. Further, Xu believed that *shipping*, which used to be a sub-culture in China, is now becoming a mainstream culture through the fast spread of new media [23].

### 2.4. Theories of Interpersonal Communication

Social interaction or interpersonal communication is one of the key components of fandom group activities [20]. Knapp proposed the model of interactive stages, including the “coming together” and “coming apart” stages of relational development [24]. The coming together stage includes initiating, experimenting, and bonding interpersonal relationships, whereas the coming apart stage includes the process of avoiding or terminating relationships [24]. The theory indicates that interpersonal relationships are not stable, which can change across time. Similarly, Baxter proposed the Relational Dialectic Theory and believes that change is the key component of interpersonal communication [25]. Given that the development of relationships can be divided into different stages, it seems necessary to consider the potential change or progress of the relationships between fans and idols or relationships between fans and fandom groups. However, it remains unclear whether such stages exist for *CP fandom* communities, and more investigation is needed.

### 2.5. Research Aims

Due to the lack of research on *CP fans* in China, a qualitative research strategy seems appropriate for the present study, which can explore the topic deeply and generate key themes [26]. This study obtained data from personal interviews; through an exploratory analysis of grounded theory, it aims to generate a comprehensive psychological dynamic development model of Chinese *CP fans*’ *shipping* behavior, which can complement and contrast preconceived ideas in theoretical construction.

## 3. Methods

### 3.1. Participants

The interviewees were recruited via snowball sampling. The first three authors used their identities as *shippers* to recruit participants through the recommendation of acquaintances and relevant communities in social media groups (such as micro-blog and QQ groups maintained by *CP fans*). The participants need to have rich practical experience in *shipping* behaviors for more than one year. Finally, the researchers identified 31 valid participants. The basic information of the participants is shown in Table 1. Only three of the respondents were male, and the rest were female.

The most representative *CP*s shipped by the participants are “DaChu” (太中) composed of the main characters Dazai Osamu and Nakahara Chuuya in the Japanese animation series *Bungou Stray Dogs* and “Eunhae” (赫海) composed of the popular Korean idol group *Super Junior* members Eunhyuk and Donghae. Both “Dachu” and “Eunhae” are non-official male/male *CP*s imagined by fans. *Official CPs* refer to the officially announced character pairings in the original works, while *unofficial CPs* are not officially recognized but are perceived by fans themselves [27]. The fan groups of these two *CP*s are large, and they have strong cultural productivity and consumption power. Other *CP*s in the interview involve characters from reality shows, e-sports, boy groups, and other domains. This study focuses on the common psychological mechanisms of these *CP fans* regardless of the particular pairings.

### 3.2. Procedure

One-to-one semi-structured interviews were used to collect data. Each interview lasted about 40 min to 60 min. The interview venue and mode were selected considering the convenience of the interviewees. Five participants were interviewed face-to-face, and twenty-six participants were interviewed via online instant teleconferencing apps such as WeChat and Zoom. Before the interviews, the interviewees were informed about the purpose of the interview and the protection of their privacy, and they all signed the informed consent. The interviews were recorded with the participants’ approval. During the interviews, interaction was non-judgmental, keeping the questions open-ended to elicit authentic and reliable responses. When the information provided by the interviewees reached a point of saturation (i.e., when few new ideas emerge), the interviewer and the interviewee jointly decided to end the interview.

### 3.3. Materials

The interview outline was initially set up by the researchers after reading the relevant literature and was revised after the pilot study (the first three interviews). The final revised interview schedule included four parts: *CP fans*’ demographic information, the reasons for *shipping*, the behaviors of *shipping* (including details of their initial *shipping* experience and later development), and the impacts of *shipping* behaviors on themselves and the fan community.

### 3.4. Data Analysis

The first author and the second author transcribed the interview texts. Data analysis was conducted using NVivo 11.0 and followed a grounded theory approach [28], and a systematic design [29] was applied. The first step of open coding was to identify, emphasize and mark meaningful units surrounding the act of *shipping*, and to give concept names until theoretical saturation. This analysis found similarities in the causes and development of *shipping* among respondents. In the second step of axial coding, the internal relations between these categories were clarified and sorted out, and the meanings reflected by these concepts were further extracted through continuous comparison and theoretical comparison, and 16 related themes were obtained. The third step was selective coding, aiming at establishing a theoretical system with internal consistency. On the basis of existing concepts, a core category that could best cover all categories and phenomena (the three stages of *shipping* development) was selected and refined to serve as a clue for research, forming a global theory and explanation. When the last three interview texts were coded, it was found that no new concepts or categories were generated, which indicates that the psychological dynamic development model of Chinese *CP fans*’ *shipping* behavior constructed through the coding process has reached saturation [30].

## 4. Results

**Figure 1:**
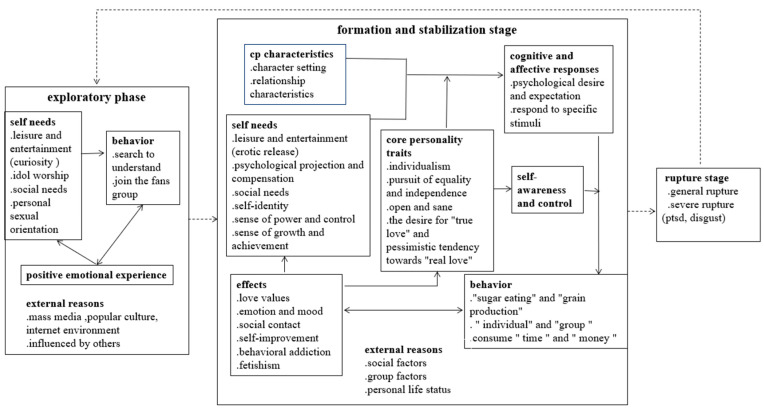
Generation, development, and consequences of *shipping*. The dotted lines indicate the phases that may be experienced.

Through the qualitative data analysis of grounded theory, we compiled the model of the formation, development, and consequences of *shipping* (see Figure 1). Table 2 shows the three categories of codes: the causes, behavior patterns, and effects of *shipping* behavior.

The development of *shipping* behavior can be divided into three stages: the exploration stage, the formation and stability stage, and the rupture stage. The *CP fans’* cognition, behavior, and consequences in each stage can be different. In the exploratory stage, external factors have a great influence on individuals. Individuals are influenced by others around them or their social and cultural environment and begin to know a certain *CP* out of curiosity, entertainment, idolatry, or other mentalities.

It is worth noting that 84% of the respondents began *shipping* in primary or junior high school. Compared with other types of *CP*s, male/male *CP*s, which are not recognized by the mainstream culture in China, can more strongly stimulate their curiosity and desire to explore. If individuals experience positive emotions, they will continue *shipping*, and with the deepening of their affection for a pairing, their *shipping* behavior will enter the *formation and stability* stage. In this stage, *CP fans* often have complex self-needs and behavior patterns and may develop *shipping* into a habit. They may respond to stimuli related to *CP fandom* like a conditioned reflex. If a fan loses interest in a pairing, their *shipping* behavior enters the stage of rupture. The following quote shows how one participant experienced all three stages:

*“I saw the video of Eunhae on Bilibili (video website) and began to love the CP. Shipping has brought me a lot of happiness and I made a lot of friends in the process. If one day one person in my CP marries someone else, I will stop shipping. Although I feel a little disappointed, I still wish them well.”* (H3)

However, not all individuals go through the three stages. For example, the last stage, *rupture*, might not appear for some.

### 4.1. Reasons for Shipping

*Shipping* is influenced by both individual factors and external factors. Individual factors are mainly self-needs and core personality.

#### 4.1.1. Individual Factors

*CP fans* can satisfy their entertainment, social, and other psychological needs by *shipping*. *Leisure and entertainment* were important motivations shown by all interviewees. Individuals can effectively relieve the pressure of life during the process of *shipping*. The content of erotic descriptions in *CP* culture releases readers’ sexual desire in a safe and covert way under the conservative environment of China, which is especially appreciated by fans.

Most of the interviewees lack experiences of maintaining an intimate relationship. They project the ideal relationship on *CP*s and compensate for their desire for romance by *shipping*. At the same time, they are also seeking their own identity. They project their ideal self onto the *CP* characters. They are *CP fans* as well as *solo fans* of one or both of the characters. In particular, some fans are *mother fans* of idols. That is, they regard idols as their own children, and their *shipping* behavior is a hope that the idol would gain happiness and personal growth through this relationship.

*Shipping* also greatly satisfies the interviewees’ social needs. They join the relevant *CP* network community in the process of *shipping*, where they share and discuss *CP* topics with other fans *shipping* the same pairings and gain a sense of belonging and group identity. In particular, creators of *CP*-related work not only seek to satisfy their imagination but also to gain a sense of growth and achievement when their works are recognized by other *CP fans*. Fans vote, buy goods, or hold festivals or other events for the idols or virtual characters involved in their loved *CP*s. This can give individuals a sense of achievement, which is, though, based on collective behavior. The following quote illustrates such social gains:

*“Shipping is for happiness. Having a relationship yourself is too much trouble. However, watching other people do this thing is fun. I also draw for my loved CP Gilgamesh/Enkidu. I published a few fanart on Lofter, and I got a lot of likes, which really gives me a great sense of achievement.”* (A)

It can grant a sense of power and control through *shipping* non-official *CP*s derived from fans’ own imaginations. In particular, male/male *CP*s enable women to shed the stereotype of females in the traditional heterosexual framework and gain the power to gaze at and manipulate men.

Apart from the desire to satisfy various needs, personality traits can also be the cause of *shipping* behaviors. Personality affects the individual’s response to different *CP*s and *shipping* behaviors through self-awareness and control. We found that the interviewees had some common personality characteristics: (1) Individualism—they choose the type of pairings and behavior pattern of *shipping* totally depending on their own preference; (2) Pursuing equality and independence—they prefer *CP* relationships where both parties are perceived as equal and independent and believe that any sexual orientation of love should be treated equally; (3) Openness—they hold an open and tolerant attitude towards various *CP* relationships and fan-created work; (4) Rationality—most interviewees can control their behavior of *shipping* and consciously abide by the rules of the fandom, and would not blindly accept what they encounter in the process of *shipping*; (5) A pessimistic perception of real love—*shipping* reflects the interviewees’ yearning for a better relationship, but most of the respondents regard the ideal true love as hard to find in reality. The following quote shows an interviewee’s pursuit of equality and a pessimistic perception of real love:

*“I like an equal and independent relationship best, and the CP Lu Guang/Chen Xiaoshi I like has such a relationship, but I haven’t seen it in real life and I don’t think I’m lucky enough to have one.”* (I)

#### 4.1.2. External Factors

External factors that affect *shipping* behaviors include the characteristics of *CP*, social factors, group factors, and personal life status.

*CP* characteristics include the traits of the relationship and character setting. For fictional characters, they have unique designs, including appearance, personality, life background, etc. For public figures in real life, they also have specific public images [31]. Whether the *CP* characteristics meet individual needs has become a key factor affecting *shipping*. The interviews found some common *CP* characteristics that *CP fans* generally like: (1) appearance—*CP*s with handsome/beautiful characters are preferred; (2) characters being single in their setting or public image; (3) novelty—the *CP* characters have complex inner minds and personalities, and their life experiences and backgrounds are unusual; (4) equally strong power—equivalence of the strong physical, spiritual, or mental power of both characters. Many interviewees regard that compared with traditional heterosexuality (in which male is usually stronger than female), such couplings can better reflect the value of equality in relationships; (5) being complementary to each other—the reciprocal matching and supplementation of the characters in appearance, personality, and ability, which shows the uniqueness of the *CP*; (6) sweetness—the relationship is generally progressing well. The universal pursuit of sweet love may reflect the anxiety and exhaustion of young people in real life, as shown in the quote below:

*“Life is so tiring, so I just want to ship someone whose relationship is sweet. Dachu is my favorite couple. Their character settings are not only interesting but complementary in every aspect. Chuuya is only 1.6 m tall, has very cute appearance, but he has the utmost force in anime; Dazai has outstanding intelligence, and is a handsome man who is 1.8 m tall. Every time I see them defeating an enemy tacitly or arguing like schoolboys, I would think they are a good match as a couple and feel really fun!”* (T4)

This quote also exemplifies the *CP* feature of being complementary to each other (point 5 above).

Social factors include popular cultural factors such as feminism, *Tanbi* (a Japanese term meaning obsession with male beauty), consumerism, and their development in the internet environment. The growth of feminism and *Tanbi* has changed the public’s preference for *shipping*, which has caused male/male *CP*s emphasizing equal relationships to become increasingly popular among the public. As for consumerism, many respondents were clearly aware that much capital investment goes behind their favorite *CP*s, and that investors may secretly demand more intimate interactions between the *CP* characters to attract more *CP fans*; for example:

*“Although I like Dachu very much, I have to admit that they are the official tool to make money. They are not couples in the anime. At the beginning, there were few scenes of them together, but there were too many Dachu CP fans, so the official continued to show their intimate interaction. Many anime products about these two characters are sold together, which is to earn money from CP fans.”* (T5)

Even if these interactions do not follow the genuine storyline (for virtual characters) or are not out of true feelings (for real persons), they allow *CP fans* and the capital force to achieve a win-win situation. Furthermore, the internet environment not only brings rich *CP* information to individuals but also provides convenience for *CP fans* to establish communities of interest and communicate with their peers. Fans can also express their views on *CP*s freely on the internet.

Fans’ interpersonal relationships in *CP fan* groups, life pressure, and personal life status could also affect *shipping* behaviors. For example, when their life pressure is too much or their interpersonal relationship is poor, interviewees reported that they would like to relieve their negative emotions through *shipping*. In addition, the individual also wants to find fun through *shipping* when bored.

### 4.2. Behaviors of Shipping

The interviews revealed various *shipping* behaviors of participants, corresponding with the behaviors in the literature. Considering cultural production and consumption, *shipping* can be roughly divided into two situations: “eating sugar” (consumption) and “grain production” (production). Considering the number of participants, *shipping* can be an individual behavior or a collective behavior. *Shipping* can also cost money and time, and the consumption situation of different individuals is different.

“Eating sugar” (*Chi-tang* in Chinese) refers to *CP fans* seeking anything (videos or pictures) that can prove or reflect the intimate relationship between their *CP* characters; “Grain production” (*Chan-liang* in Chinese) refers to the re-creation of fan art for the *CP* they like. In addition to fan fiction, it also includes *CP* relationship interpretation and character analysis. *CP fans* who can create excellent works often have a great influence on the fan group. In total, 71% of the respondents have creative behavior.

All interviewees had the experience of *shipping* with others. In total, 15 interviewees joined a network *CP* community on social media such as QQ, WeChat group, or Weibo and often interacted with others in the group; 12 interviewees joined the community only to obtain *CP* information or purchase *CP*-related items, and they hardly interacted with others; 13 interviewees only interacted with their familiar *CP fan* friends; 6 interviewees reported to prefer *shipping* alone. The following quote reflects some of these behaviors and the associated feelings:

*“My shipping behaviors are mainly watching CP interaction, editing video clips or translating foreign work for my CP Eunhae. I like many creators in fan groups! I admire that they can express their love for CP with their talents such as writing or painting, and the creation is wonderful!”* (H3)

Most interviewees chose whether to interact with other fans according to the specific situation; for example, five interviewees reported that fandom has many conflicts, and when conflict occurs, they tend to ship alone.

We asked respondents about their consumption of time and money for *shipping*, and the behaviors varied greatly. In terms of money, 4 interviewees did not spend money; 5 interviewees spent less than 300 RMB; 18 interviewees spent thousands of RMB; 3 interviewees spent tens of thousands of RMB; and 1 interviewee did not recall the specific amount but reported a willingness to spend money as long as the *CP* makes her happy. In terms of time, almost all respondents reported that they only ship when they are free and would not let the behavior affect their work or study. One interviewee only ships for 2 h per week; ten interviewees for about 1–3 h per day; six interviewees spend about 3–5 h per day; four interviewees spend about 5–7 h per day; one of the interviewees who consumes the most time spends 10 h *shipping* every day; and nine interviewees did not specify the amount of time. Notably, some participants’ reported amount of time spent on *shipping* (e.g., over 5 h per day) may contradict their statement that *shipping* did not interfere with their work, given that all the participants were undergraduate or postgraduate students.

### 4.3. Effects of Shipping

The impacts of *shipping* on individuals were mainly manifested in the formation and stabilization stage and the rupture stage. In the stage of formation and stabilization, the consequences of behavior are manifested in five aspects: love values, mood and emotion, social interaction, self-improvement, behavior addiction, and blind worship.

The influence of *shipping* behavior on individuals’ love values was multifaceted. Twelve interviewees reported that *shipping* affected their imagination of ideal love and raised the standard of love. Eleven interviewees reported that the behavior reduced their desire for love, but in two interviewees, it increased their desire for love, and what they longed for was a similar relationship to their favorite *CP*. In total, 74% of the respondents reported that their love values became more open through *shipping*; for example, individuals who used to have conservative sexual concepts could now regard premarital sex as acceptable. Except for two respondents who reported that *shipping* affected their sexual orientation, all other respondents reported that *shipping* did not affect their sexual orientation.

In terms of mood and emotion, almost all the interviewees said that *shipping* could effectively relieve their life pressure and bring pleasure to themselves. Seven interviewees mentioned negative emotional experience, which was due to reading sad fan-fictions or encountering interpersonal conflicts. Five interviewees clearly expressed their strong emotional attachment to their favorite *CPs*, and they regarded their favorite *CPs* as the spiritual pillar of their lives.

In terms of social interaction, 81% of the respondents said that *shipping* had a positive impact. However, the respondents often made friends with other fans who liked the same or similar *CP* as themselves. If other people’s views on a *CP* are quite different from theirs, the behavior of *shipping* may hinder their social interaction.

*Shipping* helps individuals to improve themselves. Ten interviewees regarded their favorite *CP* as role models and were influenced by *CP* to work hard; 78% of the interviewees said that *shipping* improved their motivation to do things; for example, they learned skills of literary creation or video production in order to create for their *CP*; and *Shipping* also enabled them to gain new knowledge.

The behavior of *shipping* has the risk of addiction, but only eight respondents mentioned this negative impact on them. It was mainly manifested as the overconsumption of time and money and damages to physical health or work/study performance. An overall positive influence of *shipping* (despite some elements of addiction) is exemplified in the following quote:

*“I made a lot of friends through shipping, and I also learned to draw in the process, and received encouragement and praise from my friends, which made me very happy. I am very envious of the relationship in my CP He Chao/Xie Yu. They encourage each other and progress together. At the same time, they are my role models, loving them makes me more diligent in study. All in all, what shipping brings to me is basically positive, except sometimes I get addicted to it and stay up late, which affects my sleep and makes my eyes hurt.”* (F)

*Shipping* can lead to “blind worship”, which includes the blind worship of people and relationships. The blind worship of people is manifested in that fans regard the characters involved in *CP*s or fans with strong cultural or economic capital as their *idols* and identify with the idols’ values and behavior patterns without distinguishing right from wrong. The blind worship of relationships can be that individuals over-identify with their favorite *CP* relationship and strive to maintain it without considering realistic factors, which may have a negative effect on the persons or characters involved in *CP*s and also the establishment of fans’ relationships in reality.

The rupture stage can be general or severe depending on the degree of *shipping*. *General rupture* is when stopping *shipping* has less impact on fans in all aspects. Fans may experience negative emotions for a period of time, but they can control the impact of emotions on life or quickly find new *CP*s to ship. *Severe disruption* is when individuals are greatly affected by stopping *shipping*. Some individuals show a certain degree of “post-traumatic stress disorder” (PTSD), such as being disgusted with *shipping* and being unable to move on to other *CP*s. This situation is often caused by greatly negative news reports or poor handling from *CP* characters (usually real persons). Only four interviewees in the interview had a severe rupture, which though seemed highly impactful, for example:

*“I used to like the CP Bo jun yi xiao, but later I stopped shipping because of the 227 incident (a viral confrontation on the Internet due to a fan fiction on this CP, mentioned earlier). I was afraid of cyber violence. During that time, I felt very frightened when I watched fans quarreling. I also uninstalled Weibo for a period of time. In short, this incident left me some deep psychological trauma.”* (E)

## 5. Discussion

### 5.1. Summary of the Findings

Based on the systematic analysis of interview data, this study established a dynamic development mechanism of *CP fans*’ *shipping* behavior. *CP fans*’ love for a pairing may go through the *exploratory stage*, *formation and stability stage*, and *rupture stage*, although not all individuals would necessarily go through all three stages. Entertainment and social interaction needs, personality traits, and external factors (e.g., fan groups) are the main factors of *shipping*. These lead to the development of *shipping* behaviors, including *CP* consumption versus production, shipping alone versus shipping in groups, and spending time and money. The effects of *shipping* include emotional gains, changes in love values, and self-improvement, but also blind worship, behavioral addiction, and rupture.

### 5.2. Implications

#### 5.2.1. Self-Identity in Realistic Dilemma

The study found that the *shipping* of young people in the Z generation is a way to alleviate their self-identity crisis in the internet environment. Self-identity can be understood as an individual’s self-evaluation and self-positioning in terms of occupation, politics, religion, and values [32]. Most of the respondents in this study were young people aged 18–30 who had few experiences of relationships or had poor relationships; 90% of them were women, and 35% were sexual minorities. At present, China is in a period of rapid economic development. The competitive social environment and pluralistic values make young people easily fall into anxiety and confusion about the future. The awakening of women’s independent consciousness and the openness and advancement of young people’s minds are in strong conflict with the long-held constraints and stereotypes of women and sexual minorities in the patriarchal society. These factors make the young generation easily fall into a self-identity crisis. They have uncertainty about themselves and their future.

Unlike the simple distinction between *CP fans* and *solo fans* in previous Chinese literature [27], this paper finds that many interviewees were not only *CP fans* but also fans of one or both *CP* characters. They project their ideal self onto their *CP*, seek appropriate positioning for their outlook on love and life, and construct their own identity in the process of *shipping*. To a certain extent, this alleviates the individual’s self-identity crisis in reality. All the actions in *shipping*, such as joining *CP* communities, creating fan art for *CP*, and purchasing *CP*-related goods, are the construction and manifestation of their identity as *CP fans*.

#### 5.2.2. Group Interaction under the Effect of Individualization

The individualization theory holds that people would like to “live for themselves” [11]. Yan defined subjective individualization as people’s strong willingness to achieve their goals in education or career and satisfy their emotional or physiological desire in private life [12]. This study found that individuals’ *shipping* behavior showed a strong individualization characteristic. *CP* fans put their feelings in first place and pay more attention to their own experience in the process of *shipping* than to the group identity (although group identity is still important). Consistent with the conclusions of previous literature [3], the *CP* fans in our study relied on the network to establish communities, distinguish well from non-fan groups through the use of specific cultural terms and rules, and gain a sense of belonging and identity in the process of interaction with members. However, this study argues that individuals’ desires to obtain a group identity may narrow their outlook. Most of our participants were not open to all fandoms of a certain *CP*. They did not like to *ship* in a large fan group where opinions are likely to diverge but only interacted with a few peers to avoid negative emotional experiences.

From the interviewees’ accounts, conflicts seem to be ubiquitous in fandoms. Previous literature focuses on the conflict between *CP fans* and *solo fans* and uses *in-group preference* to explain the group conflict [16,33]. This study found that conflicts may break out between different *CP fan* groups and within the same group, and the essential reason for the conflict is fans’ different viewpoints. The openness and anonymity of the network increase the possibility of the collision of different views. The individualization characteristics of fans make them more inclined to maintain their own views. The prevalence of younger-age fans nowadays is also one of the factors affecting the frequent occurrence of conflicts. As minors have limited knowledge and are less mature, they tend to view things from a narrow perspective, so they are prone to conflicts with others under the influence of emotions and groups. In sum, this study concludes that holding different ideas is the main reason for the conflict in fandoms; the individualization of fans, in-group preference, network influence, and the younger age tendency of fans are important factors causing the conflicts.

#### 5.2.3. Immersive Cultural Consumption

As a type of cultural consumption, there is a lack of research on the experience of *shipping* in the previous literature. Life in a *consumer society* is becoming increasingly symbolic, and people are no longer simply consuming commodities but consuming the symbolic value endowed by commodities [34]. *CP fans* pursue not only *CP* itself but also the ideal intimate relationship represented by the *CP*. Fans trade with capital by purchasing *CP*-related goods in order to keep updated with *CP* interaction [35,36]. In addition, *CP fans* are not passive recipients of this culture, but active participants and producers of culture. Around the *CP*s’ image in mass media, *CP fans* create a large number of cultural texts and other art forms, which embodies the desire and ability of fans to participate in cultural production [4].

This study argues that the reason why the audience continues to *ship* is that they receive immersion experiences in the process. Immersion experience is the core concept of immersion theory, which refers to a kind of emotional experience in which people are very interested in and fully engaged in a certain activity [37]. There are several factors associated with such an immersive experience, including a personal need for pleasant feelings, group identity, and a sense of achievement. *Shipping* meets individuals’ various internal needs, and the behavior itself is the purpose. When an individual ships the *CP* completely according to their own preferences, the individual experiences a complete sense of control. When they use network technology to search for *CP* information and consume traces of *CP* interaction, mirror neurons in the brain will be activated, releasing signals of pleasure [38]. Interaction with peers can enable individuals to gain a sense of group identity, and their pleasure will be magnified by group influence. Further, through the demanding task of creating artwork for *CP*s, fans feel the satisfaction of creativity and imagination; when the works are recognized by others, they experience a sense of achievement.

#### 5.2.4. A Double-Edged Sword of Emotional Games

In essence, *shipping* is an illusory emotional game among fans created by capital forces, in order to cater to the psychological needs of the public in the consumerist environment. Although this game alleviates the loneliness and uneasiness of contemporary youth to a certain extent, it may also bring many adverse effects to their real life.

The competitive social environment means young people have little time to take care of their emotional life, and some individuals can only seek ideal relationships from the illusory *CP* world constructed by capital forces. Through analyzing popular *CP* characteristics, it was found that young people, especially women, have an independent and enterprising spirit despite their tiresome living conditions. They like the relationship between two equally strong persons, showing equality and independence, hoping that this relationship can bring emotional comfort to themselves as well as help their personal development. The preference for male/male *CP*s reflects their dissatisfaction with the dependent status of women in the traditional heterosexual framework.

On the other hand, *shipping* may also cause many negative effects. For example, because the imagined ideal relationships are often inconsistent with the reality, long-term *shipping* may cause individuals to have unrealistic love assumptions and hinder the establishment of intimate relationships in real life. Excessive *shipping* may also affect individuals’ work and study. In this study, the respondents described little about the negative impact of *shipping*, which may be due to the strong self-control ability of the respondents. It may also be a social approval effect, in that participants may reduce the negative description of themselves in interviews.

#### 5.2.5. Different Stages of *CP fandom* Behaviors

The present study identified three stages of *shipping* behaviors: the exploration stage, the formation and stability stage, and the rupture stage. This finding could be explained by the theoretical model of interactive stages proposed by Knapp [24] and the Relational Dialectic Theory by Baxter [25]. The relationship between *CP fans* and their *CPs,* as well as the fan group, appears to be unstable as interpersonal communication or relationships can always be changeable, with close or distant relations (as suggested in the theories). The theories explain why *CP fandom* behaviors can be unpredictable and unexpected, as *CP fans* might quickly switch between different stages when shipping one particular pairing. For example, emotional gains and trust in the *CP* can turn into rupture when negative events happen. The sense of belonging to fan groups can also end when conflicts appear within and between groups.

### 5.3. Limitations and Future Directions

As an exploratory study, this paper summarizes the psychological and behavioral mechanism of *shipping* through grounded theory. It also has some shortcomings and areas for improvement and development. First, for the interviewees, because the researchers recruited the participants based on their fan status, their preference for *CP* and demographic variables such as educational level may be highly homogeneous, which limits the representativeness of the sample and the generalizability of results. Future research can further expand the richness of the sample. Secondly, the data obtained from the interviews in this study were recall data from the participants, which were not timely. Future research can be combined with experimental methods, such as daily diary reports, to improve the timeliness of findings. Finally, this study used qualitative research methods to construct a basic theoretical framework, but the description of the development trajectory of *shipping* could be more detailed. Based on this study, more quantitative studies can be designed to verify the role of various factors at different stages of development. For example, questionnaire surveys could be distributed among *CP fans* to explore the relationship between the levels of engagement in *shipping* and individual or environmental factors.

## 6. Conclusions

As a unique idolatry behavior, *shipping* brings pleasant emotions and better social interaction to *CP fans*, but also negative feelings and social conflicts. The reasons for *shipping* can be complex and include both individual (e.g., social needs, psychological projection, and compensation) and external factors (e.g., pop culture and complex internet environment). Individuals need to be aware that the *CP* images presented by the media can be illusory after all. *Shipping*, in some cases, can only alleviate *CP fans*’ temporary emotional emptiness, but cannot bring real benefits to them. However, it is not appropriate to hold prejudice against the *CP fans* or their community, as *CP fandom* is a reasonable social-cultural phenomenon resulting from young people’s personal reassurance and social needs, or the changing social environment. Individuals may begin, develop, or end their *shipping* experience as their life experiences change.

## Figures and Tables

**Table 1:** Basic information of interviewees.

No.	Sex	Age	Level of Education	Sexual Orientation	Marital or Emotional Status	Favorite *CP*	Initial *Shipping* Time
**A**	F	18	Undergraduate	Not sure.	Single	Gilgamesh/Enkidu (anime *Fate* roles)	Primary school
**B**	F	19	Undergraduate	B	Single	ChanBaek (idol group *EXO* members)	Primary school
**C**	F	19	Undergraduate	H	In love	Fu Sichao/Xv Yang (variety show *the coming one* players)	Primary school
**D**	F	20	Undergraduate	H	In love	Tian/knight9 (*League of Legends* professional players)	Primary school
**E**	F	24	Graduate student	H	Single	Ship widely	Primary school
**F**	F	18	Undergraduate	H	Single	He Chao/Xie Yu (novel *Camouflage scum* characters)	Twelfth grade
**G**	F	23	Graduate student	H	Single	Even/Isak (TV drama *Skam* characters)	Senior
**I**	F	20	Undergraduate	B	Single	Lu Guang/Chen Xiaoshi (Anime *Link click* Characters)	Primary school
**J**	M	18	Undergraduate	G	Single	Song Yaxuan/Liu Yaowen (idol group *Teen in Times* members)	Primary school
**K**	M	18	Undergraduate	H	Single	Ship widely	Primary school
**L**	M	21	Undergraduate	G	In love	Gu Hai/Bai Luoyin (TV drama *Overdose* roles)	Primary school
**T1**	F	18	Undergraduate	H	Single	DaChu	High school
**T2**	F	20	Undergraduate	Not sure.	Single	DaChu	Freshman
**T3**	F	24	Graduate student	H	Single	DaChu	Senior
**T4**	F	18	Undergraduate	B	Single	DaChu	Primary school
**T5**	F	20	Undergraduate	H	Single	DaChu	Junior high
**T6**	F	20	Undergraduate	H	Single	DaChu	High school
**H1**	F	24	Graduate student	B	Single	Eunhae	Primary school
**H2**	F	25	Undergraduate	H	Single	Eunhae	A year ago (before the interview)
**H3**	F	21	Undergraduate	H	Single	Eunhae	Junior high
**H4**	F	30	Junior college	H	Married with children	Eunhae	Junior high
**H5**	F	30+	Undergraduate	H	Married	Eunhae	Senior 2
**H6**	F	25	Junior college	B	Single	Eunhae	Five years ago
**H7**	F	19	Undergraduate	H	Single	Eunhae	Junior high
**H8**	F	19	Undergraduate	B	Single	Eunhae	Senior 2
**H9**	F	23	Undergraduate	H	Single	Eunhae	Two years ago
**H10**	F	19	Undergraduate	H	Single	Eunhae	Junior high
**H11**	F	19	Undergraduate	B	Single	Eunhae	Primary school
**H12**	F	18	Undergraduate	B	Single	Eunhae	Two years ago
**H13**	F	19	Undergraduate	H	Single	Eunhae	Senior 1
**H14**	F	19	Undergraduate	B	Single	Eunhae	Junior high

“F” = female, “M” = male, “H” = heterosexuality, “B” = Bisexuality, “G” = gay.

**Table 2:** Three categories of *shipping* development.

Main Categories	Sub-Categories	Macrothemes	Themes	Reference Point
**Reasons for *shipping***	individual factors	psychological projection and compensation	spiritual and emotional sustenance; individual sexual orientation	40
		self-identity	identify with feelings; identify with the role (idolatry)	55
		leisure and entertainment demand	pressure release; release of lust; curiosity and voyeurism; interests and hobbies; beauty consumption; satisfaction of imagination; psychological safety	162
		social needs	avoid loneliness; sense of belonging and group identity; group craze	71
		special psychological needs (formation and stabilization stage)	pursuing a sense of power and control; gaining a sense of growth and accomplishment	52
		other individual factors (formation and stabilization stage)	core characteristics of personality	102
	external factors	exploratory phase’s external causes	mass media; pop culture; internet environment; influence of others	77
		formation and stabilization stage’s external causes	*CP* characteristics; social factors; group factors; personal life status	272
**Behavior of *shipping***	exploratory phase‘s behavior	search to understand, join the fans group		46
	formation and stabilization phase behavior	“eating sugar” and “producing grain”; “individual” and “group”; consume time and money		170
**Effects of *shipping***	impacts during the exploratory phase	positive emotional experience		315
	impacts during the formation and stabilization stage	love values	an idealized vision of love; affect love desire; a more open view of love; affect one’s own sexual orientation	50
		mood and emotion	emotional experience; emotional attachment	43
		social intercourse	expand the scope of social interaction; improve social skills and deepen friendships; avoid social interaction; conflict	102
		self-improvement	role model; incentive effect; expand the scope of knowledge	65
		behavioral addiction	spend a lot of time and money; affect study and work; damage to the body	8
		blind worship	blind worship of man; blind worship of relationship	11
	impacts during the rupture phase	general rupture performance	negative emotions; looking for a new *CP*	28
		severe rupture performance	post-traumatic stress disorder (PTSD); disgust	9

## Data Availability

The data are not publicly available due to privacy.
